# In memoriam: Charles Boucher (1958‐2021)

**DOI:** 10.1002/jia2.25709

**Published:** 2021-05-01

**Authors:** Annette H Sohn, Jonathan Schapiro, Peter Reiss

**Affiliations:** ^1^ TREAT Asia amfAR – The Foundation for AIDS Research Bangkok Thailand; ^2^ National Hemophilia Center Sheba Medical Center Tel Aviv Israel; ^3^ Department of Global Health Amsterdam University Medical Centers University of Amsterdam Amsterdam Institute for Global Health and Development, and HIV Monitoring Foundation Amsterdam The Netherlands

*Dr*. *Charles Boucher*

*Scientific* *Director, Virology Education and Academic Medical Education*

*Professor,* *Erasmus Medical* *Center, Rotterdam*

We were saddened to learn of the passing of Dr. Charles Boucher on 26 February at his home in Utrecht, The Netherlands (Figure 1; [1, 2, 3]). Dr. Boucher was a highly respected clinician, researcher, mentor and educator, who importantly contributed to advancing the field of HIV through his scientific curiosity and passion for training and capacity development.

**Figure 1:**
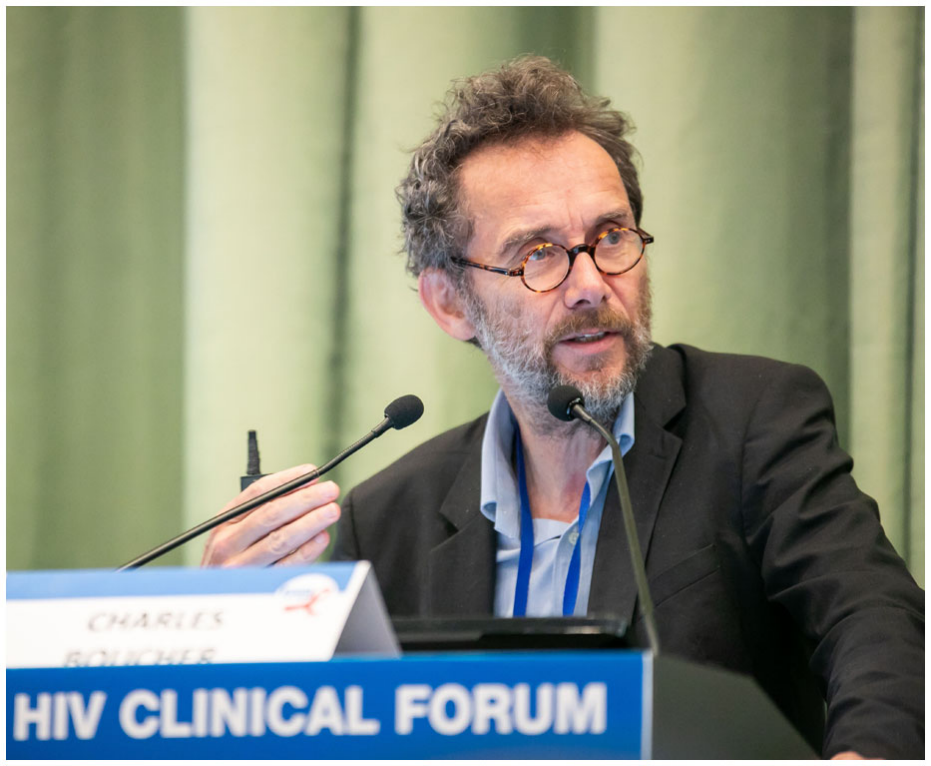
Photo of Dr. Charles Boucher, courtesy of Virology Education.

He is perhaps best known for his early contributions to HIV resistance research and efforts to translate basic and laboratory science into improvements in the quality of care and treatment. Dr. Boucher’s groundbreaking work three decades ago laid the foundation of much of today’s understanding of viral drug resistance [4, 5, 6]. Although originally described for HIV, it provided important insights for other viral infections, such as hepatitis C, as well as the current COVID‐19 pandemic. He was among the pioneers in describing the selection of specific mutations by HIV, how they impact viral replication and fitness, and their contribution to clinical progression. Although he worked on early HIV drugs like zidovudine and lamivudine, his observations led to a better understanding of all our subsequent antiretrovirals [7, 8]. Dr. Boucher’s insights provided scientific guidance for many of our improvements in diagnosing and monitoring resistance, as well as designing drugs with superior resistance characteristics.

Many may not be as aware of the foundational role he played in expanding international training opportunities and research workshops to topics and geographical areas that often were not the main focus of global HIV conferences. In 1998, Dr. Boucher helped to found Virology Education, an organization that institutionalized these “niche” programmes that combined clinical training with abstract‐driven research and state‐of‐the art symposia. While they started with HIV research workshops on topics like drug resistance, Dr. Boucher continued to improve the model for how to deliver these workshops efficiently and effectively to build a portfolio of over 40 annual workshops with global representation from over 120 countries. Even while he was receiving chemotherapy for pancreatic cancer last year, he was contributing to new programmes on various aspects of COVID‐19, including vaccine development, delivery and implementation.

At the same time, it is important to recognize that the scope of his work was itself also a form of advocacy. The topics that Dr. Boucher chose to build programmes around often were ones that had not been deemed of sufficient interest with large enough audiences to merit global attention. This included workshops on HIV and ageing, paediatrics, transgender people and women. There have been clinical forums for nurses, pharmacists and physicians, held in Brazil, China, Russia and other countries.

It is hard to know how many thousands of people have been impacted in some way by the programmes Dr. Boucher established and the online materials he ensured open access to, or through the improvements in the quality of care that resulted from putting data‐driven evidence into the hands of those delivering treatment. He did all of this with seemingly endless energy, and an uncanny ability to travel around the world multiple times over every year. We thank him for applying his ever‐curious mind and passion to finding more ways to advance the application of knowledge and data to improve the lives of people living with HIV around the world.

He will be greatly missed.

## Competing interests

Dr. Sohn receives grant and training funding to her institution from ViiV Healthcare. Dr. Schapiro has received research support, honorarium or consulting fees from Abbvie, Merck, Gilead Sciences, GlaxoSmithKline, Tibotec‐Janssen, Teva, Virology Education and ViiV Healthcare. He has received travel support and stipends for advisory work for the World Health Organization. Dr. Reiss, through his institution, has received independent scientific grant support from Gilead Sciences, ViiV Healthcare, Merck & Co and Janssen Pharmaceuticals Inc., and has served on scientific advisory boards for Gilead Sciences, ViiV Healthcare and Merck & Co, for which his institution has received remuneration.

## Authors’ contributions

All authors have contributed to the preparation of the manuscript, read and approved the final draft.
